# Genetic variations in Turkey cultivar and ecotype *Medicago sativa* species: cytological, total protein profile, and molecular characterization

**DOI:** 10.1186/s43141-021-00159-6

**Published:** 2021-04-29

**Authors:** Büşra Yazıcılar, Gholamreza Jannati, Ismail Bezirganoglu

**Affiliations:** 1 Department of Molecular Biology and Genetics, Erzurum Technical University, 25050 Erzurum, Turkey; 2 Department of Agronomy, Faculty of Agriculture, Ataturk University, Erzurum, Turkey

**Keywords:** *Alfalfa*, SSR, Genetic variations, Flow cytometry, Chromosome number

## Abstract

**Background:**

*Alfalfa* **(***Medicago sativa* L.) is a perennial plant, which is high in nutritional value and resistant to environmental conditions, and it is one of most frequently preferred feed crop among the leguminous family. In this study, it was aimed to determine the genetic diversity of some *alfalfa* ecotypes and their varieties by DNA, protein, nucleus, and chromosome counts. The genetic distance between the populations of control (*M*. *truncatula*), five different cultivars (*Alsancak*, *Bilensoy*, *Iside*, *Plato*, *Bilensoy82*), and three different ecotypes (*Erzurum*, *Muş*, and *Konya*) was investigated by cytogenetic analysis, flow cytometry, simple sequence repeats (SSR), and SDS PAGE techniques.

**Results:**

Cytogenetic analysis of these tested plants has verified the existence of expected levels such as diploid, triploid, and tetraploid as well as aneuploid (*2n = 4x = 30*) plants. Flow cytometry analysis have displayed that all of tested plants were tetraploid, whereas cytological analysis had either diploid, triploid, or tetraploid. Genetic diversity dendrogram was created using *Erzurum*, *Muş*, *Konya*, *Bilensoy82*, *Alsancak*, and *Plato* varieties. The *Iside* and *Bilensoy* were found to be morphogenetic in relationship. Our control plant, *M*. *truncatula*, did not have a similarity relationship with other ecotypes and cultivars. The total numbers of protein bands differed among tested plants from 140 kDA to 25 kDa.

**Conclusions:**

This paper first reports on the genetic variation of Turkish *alfalfa* plants by using detailed analysis techniques. This work provides important findings for the classification, conservation, and innovation of *alfalfa* germplasm resources.

## Background

*Alfalfa* is an important perennial and cross-pollinated forage crop of the family *Fabaceae* grown all over the world [1]. It has a rich and long history and is one of the earliest crops domesticated by humans [2]. *Alfalfa* is the main forage crop for dairy producers of many agricultural regions due to its high feeding value. *Alfalfa* is cultivated for its high yield and environment adaptation, wide disease tolerance, and nutritional feeding quality, as well as its role on nitrogen fixation, wildlife habitat, soil conservation, and bioremediation. *Medicago* species have a wide natural distribution that covers Asia, Europe, and Africa [3, 4]. Its conventional classification is based on seed structures and other important characteristics including leaves, cotyledons, ploidy level, organ hairness, and growth habit [5]. The cultivated ones include diploid and tetraploid forms of *M*. *sativa* L., *M*. *sativa subsp*. *sativa*, *M*. *sativa subsp*. *falcata*, and *M*. *sativa subsp*. *glutinosa* [5], which are all perennial plants. *Medicago* species are either diploids (*2n = 2x = 16*), tetraploids (*2n = 4x = 32*), or hexaploids (*2n = 6x = 48*). *Alfalfa* is the most polymorphic species, which is a trait of great importance in taxonomic studies. Taxonomy depends mostly on morphology to discriminate groups. Morphological features are widely characterized by flower colors and pod shapes. *M*. *sativa subsp*. *sativa* has purple color flowers and coiled pods and is less cold resistant than *M*. *sativa subsp*. *falcata* or yellow sickle shaped pods and their hybrid progeny with mixed color flowers and partially coiled pods. The perennial *Medicago* species have a basic chromosome number of *x=8* [6]. These species are both diploid and tetraploid, self-crossed, and much more heterozygous. They are also less resistant against abiotic and biotic stress factors. The genetic variation of *alfalfa* has been determined using morphological features [7], protein markers [8], chromosome number, and molecular markers consisting of RAPDs [9], AFLPs [10, 11], and simple-sequence repeat markers (SSRs) [1, 12]. Flow cytometry is a useful method of plant varieties that identifies ploidy level and analyzes the chromosome number. It has been characterized as a rapid tool for assessing the ploidy level of *alfalfa* accessions [13]. Chromosome number analysis has been evident to be an important tool for classification and releasing chromosome origin events. The basic principle of cytological analysis is to determine classification and assessment of basic chromosome number of closely related species or other related ones. SSR markers have been used extensively for the development of new varieties diversity studies. Simple sequence repeat (SSR) markers are highly reproducible, co-dominantly inherited, and abundant markers in genomes that can be analyzed using PCR-based techniques. They have provided species-specific allele patterns in *alfalfa* and can be useful markers for determining the genetic variation and the differences between plant varieties [14]. It has been shown that SSR can be used to identify genetic variations and to characterize the genetic similarities among cultivars in *alfalfa*. Although agronomic traits methods have provided significant successes in taxonomic studies, these methods have still some restrictions because of closely related populations and species for germplasm. Molecular marker and cytogenetics techniques can considerably provide more information about chromosome number, genome size, and base pair. The aim of this study is to evaluate the genetic diversity and ploidy levels by using SSR marker, SDS PAGE, chromosome number, and flow cytometry analysis of eight *alfalfa* varieties. Moreover, it is also thought to be an important material for the improvement of new cultivars through breeding studies in the future.

## Methods

### Plant material

Control (*M*. *truncatula*); five *alfalfa* cultivars, *Bilensoy*, *Bilensoy82*, *Alsancak*, *Plato*, and *Iside*; and three *alfalfa* ecotypes, *Erzurum*, *Konya*, and *Muş* were used as the plant materials for this study. All cultivars used were obtained from the Department of Molecular Biology and Genetics at Kafkas University. Five *alfalfa* cultivars were registered by the East Anatolia Agricultural Research Institute Erzurum, Turkey.

All ecotypes used were provided from local farmers of *Erzurum*, *Muş*, and *Konya* cities. *Alfalfa* seeds were obtained in water agar. They were maintained at 25/27 °C (day/night) growth chamber with a 16 h day length for 8 days to initiate seedling. After 2 weeks, the leaves of plants were collected from cultivars and ecotypes for further analysis.

### Chromosome counting

In the mitotic chromosome analysis, 10 *alfalfa* seeds were planted in each petri dishes. After 3 days, 1.5 cm roots were cut with a scalpel. It was stored in 0.05% colchicine in glass tubes at room temperature for approximately 3 h. It was then left to wash for 3 h. Then, it was left in fixative (3:1, ethanol: 45% acetic acid) for 3 h at room temperature. Then, it was kept in a 60 °C bath for 10 min in 1 N NaOH in hydrolysis. Hematoxylin was kept at room temperature for 10 h. It is then passed through dh2O 3 times. Zeiss Axiophot microscope was used and samples were analyzed in three replicates [15].

### Flow cytometry

Nuclear DNA content of *alfalfa* varieties were analyzed using fresh plant materials by flow cytometry. Genome size analysis was determined by using 3 replicates. Commercial kits (CyStain PI absolute P) of Partec were used in nuclear DNA content analysis. A slightly modified version of the Partec protocol was carried out in the analyses. *Hordeum vulgare* “Cervoise” (2 pg/2C) was obtained as a reference standard. Shortly, the protocol consisted of simultaneously chopping leaf tissues (20 mg each) of *Alfalfa* and *Hordeum vulgare* “Cervoise” as a control in 0.5 mL nuclei extraction buffer, transferring homogenized tissues into centrifuge tubes through filter, brief centrifugation (20 s), dissolving the pellet in extraction buffer (0.5 mL), adding staining buffer (1 mL), and incubation (30 min) at room temperature. The samples were then analyzed using a Partec CyFlow Space flow cytometer (Munster, Germany) equipped with green laser excitation at 488 nm. The absolute DNA contents of *Alfalfa* varieties were calculated based on the ratios of the G1 peak means of sample and reference standard by using the following formula:

$$ \mathrm{Sample}\ 2\mathrm{C}\ \mathrm{DNA}\ \mathrm{content}=\frac{\mathrm{sample}\ \mathrm{G}1\ \mathrm{peak}\ \mathrm{mean}}{standard\ G1\  mean}\mathrm{x}2\mathrm{C}\ \mathrm{DNA}\ \mathrm{content}\ \left(\mathrm{pg}\right) $$

Nuclear DNA content values were calculated as pg by using formulas. The *c* values of the species were compared using *t* test.

### SSR

Total genomic DNA was purified following CTAB method with slight modifications [16]. Eleven primer pairs detecting SSR loci in *M*. *sativa* L. as described by Erayman et al. [17] were used in the amplification reactions. PCR reactions were applied in a 50-μl volume consisting of (200 ng) 1 μL genomic DNA, 4 μL of dNTPs, 5 μL of 10x buffer, 0.5 μL BioVan Taq pol (5 U), 1 μL each primer, and 37.5 μL of ddH_2_O. The cycling conditions for the PCR reaction were as follows: 95 °C for 45 s, 58 °C for 45 s, 72 °C for 1 min, and 72 °C final extension for 30 cycles. The PCR products were electrophoresed and visualized using a 1% (w/v) agarose gel.

### Total proteins analysis

For *alfalfa* total protein isolation, 0.03 g of leaf sample was weighed. It was taken into 2 mL Eppendorf tube and homogenized by adding 200 μL sample buffer sample. It was kept in a water bath at 100 °C for 3 min. It was then centrifuged at 10,000 rpm for 5 min. The supernatant portion was transferred to another 2 mL Eppendorf tube. Later, a standard graphic was created using the Bradford method (Table 1). In the equation created in the standard graph, the values of the protein samples are in the ordinate part (*M*. *truncatula* 0.6105, *Bilensoy* 0.6048, *Iside* 0.5181, *Erzurum* 0.6065, *Muş* 0.6316, *Konya* 0.6107, *Bilensoy82* 0.5222, *Plato* 0.4519, *Alsancak* 0.5053). Twenty micrograms of the protein was separated in 10% sodium dodecyl sulfate [18].

**Table 1:**
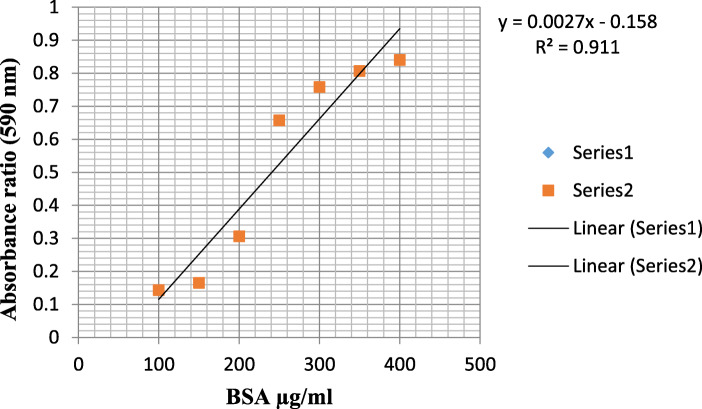
Standard plot of Bradford protein

### Statistical analysis

The data were scored as presence of band (1) and absence of band (0) for the SSR results. To identify the genetic diversity, the observed number of alleles (na), effective number of alleles (ne), Nei’s gene diversity (he), and Shannon’s information index (I) were calculated using POPGENE v1.31 [19]. A relation of similarity between varieties was calculated according to Nei (1973). A UPGMA tree was constructed using NTSYS v2.02 [20].

## Results

### Chromosome number and ploidy level

**Fig. 1:**
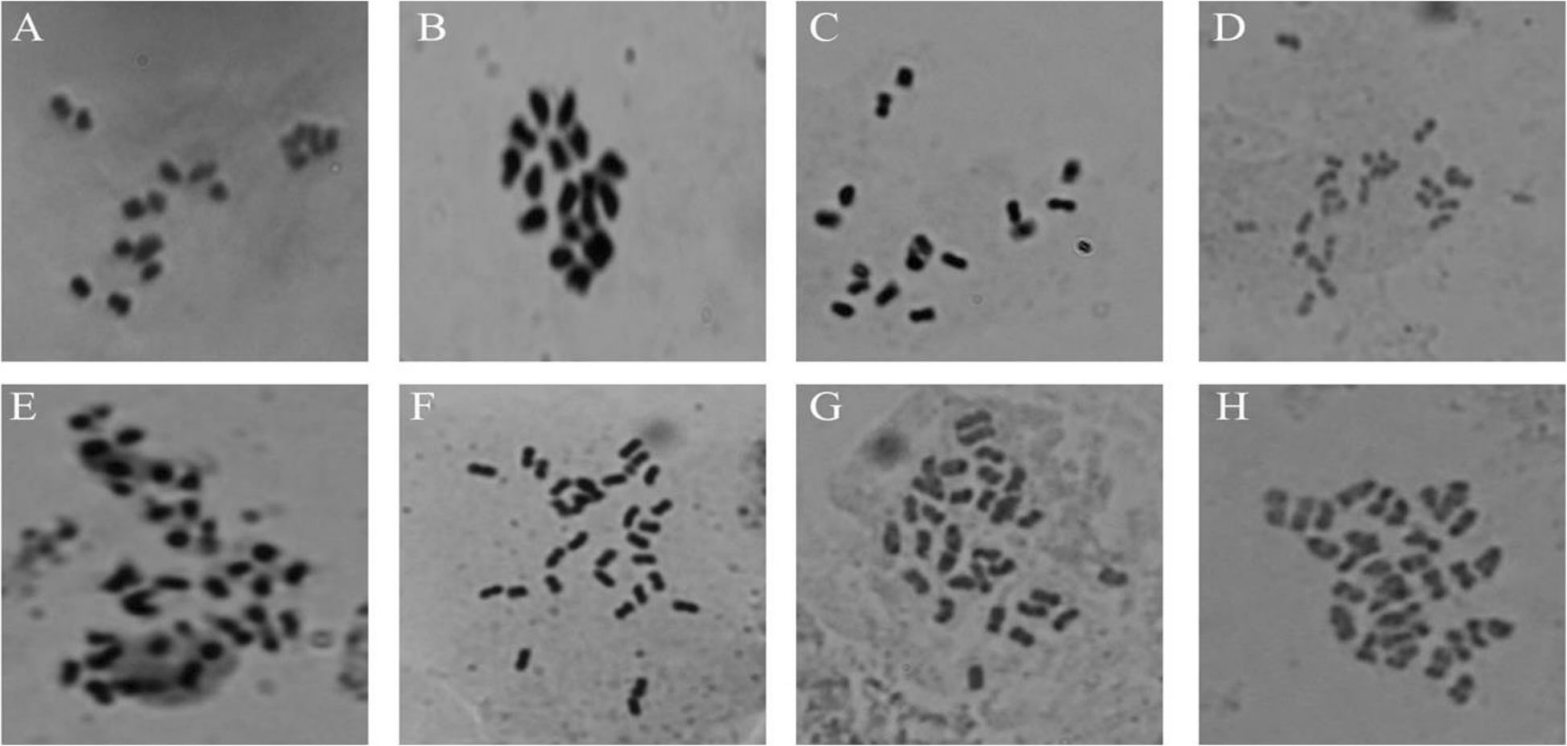
Chromosome numbers analysis of alfalfa varieties using microscopy. Representative of the ploidy levels of varieties. **a** Iside 2n = 2x = 16. **b** Muş 2n = 2x = 16. **c** Bilensoy 2n = 2x = 16. **d** Bilensoy82 2n = 3x = 24. **e** Alsancak 2n = 4x = 32. **f** Plato 2n = 4x = 32. **g** Erzurum 2n = 4x = 30. **h** Konya 2n = 4x = 30

Of eight varieties tested, the three varieties were found to be entirely diploid with basic chromosome number *2n = 2x = 16* (*Iside*, *Bilensoy*, and *Muş*). Only *Bilensoy82* was found to be triploid with basic chromosome number *2n = 3x = 24*. Four varieties were found to be tetraploid (*Alsancak*, *Plato*, *Erzurum*, and *Konya*) with basic chromosome number *2n = 4x = 32*. Although *Erzurum* and *Konya* varieties were detected to be tetraploid, basic chromosome numbers were detected as *2n = 4x = 30* (Fig. 1).

### Nuclear DNA content

**Fig. 2:**
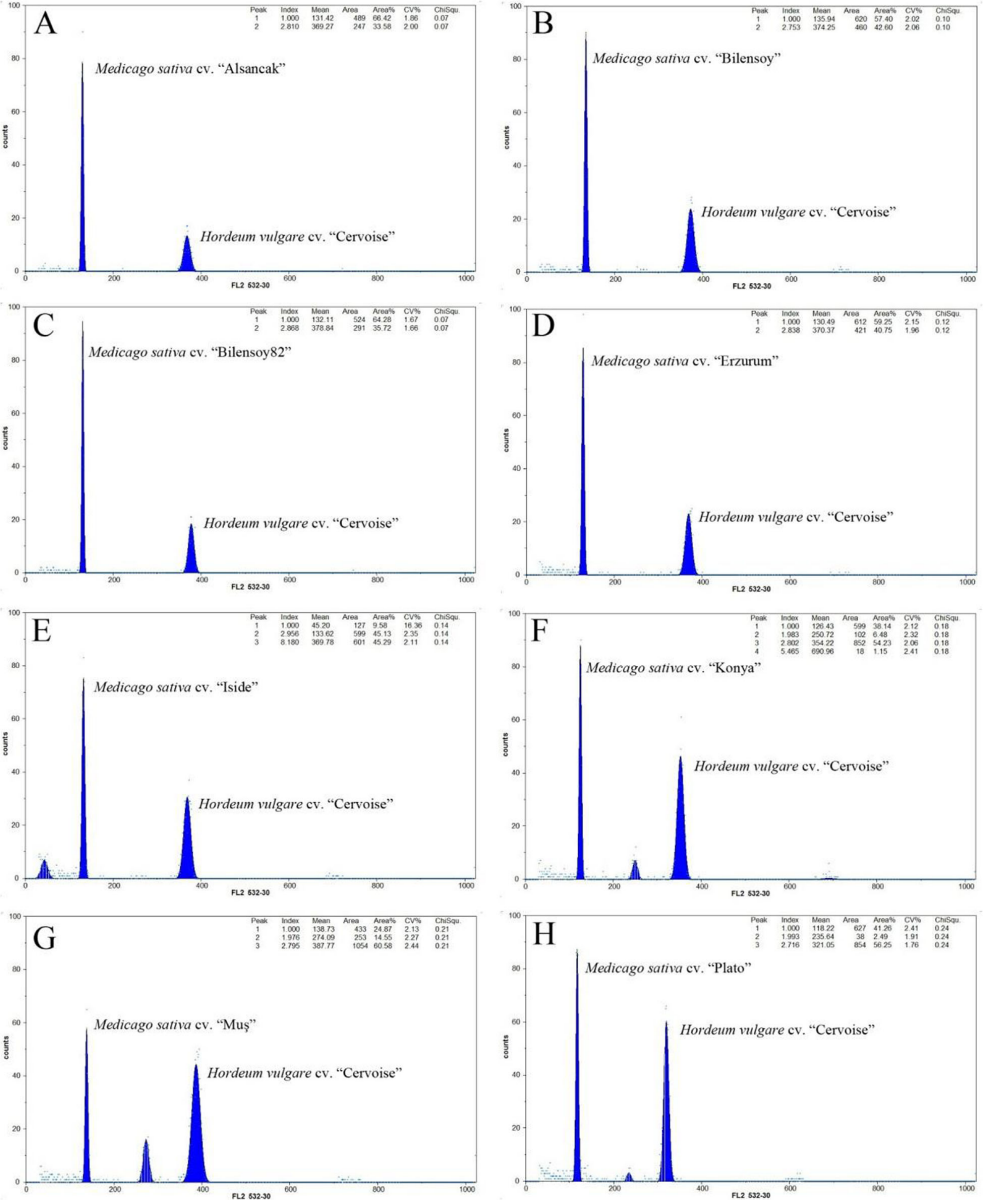
Flow cytometric analysis of PI stained nuclei *alfalfa* using *Hordeum vulgare* “Cervoise” as control. **a** *Alsancak*, **b** *Bilensoy*, **c** *Bilensoy82*, **d** *Erzurum*, **e** *Iside*, **f** *Konya*, **g** *Muş*, **h** *Plato*

The nuclear DNA content of the tested varieties ranged from 3.71 pg in *Bilensoy82* to 3.92 pg in *Plato*, which is a tetraploid *2n = 4x = 32*. Nuclear DNA content was 3.81 pg for *Muş*, 3.80 pg for *Konya*, and 3.75 pg for *Erzurum*, respectively, whereas the *Alsancak*, *Iside*, and *Bilensoy* cultivars exhibited a value of 3.79 pg, 3.85 pg, and 3.87 pg. There was no intraspecific diversity in nuclear DNA content among tested. Overall, the tested varieties demonstrated a small different value of nuclear DNA content (Table 2, Fig. 2).

**Table 2:** Nuclear DNA content of alfalfa varieties

***Alfalfa***	Sample peak	Standard peak	Standard DNA content (pg)	Örnek DNA content (pg)	CV1 (***Alfalfa***)	CV2 (***Hordum vulgare*** “Cervoise”)
***Alsancak***	131.42	369.27	10.65	**3.79**	1.86	2
***Bilensoy82***	132.11	378.84	10.65	**3.71**	1.67	1.66
***Bilensoy***	135.94	374.25	10.65	**3.87**	2.02	2.06
***Erzurum***	130.49	370.37	10.65	**3.75**	2.15	1.96
***İside***	133.62	369.78	10.65	**3.85**	2.35	2.11
***Konya***	126.43	354.22	10.65	**3.80**	2.12	2.06
***Muş***	138.73	387.77	10.65	**3.81**	2.13	2.44
***Plato***	118.22	321.05	10.65	**3.92**	2.41	1.76

### Marker analysis

**Fig. 3:**
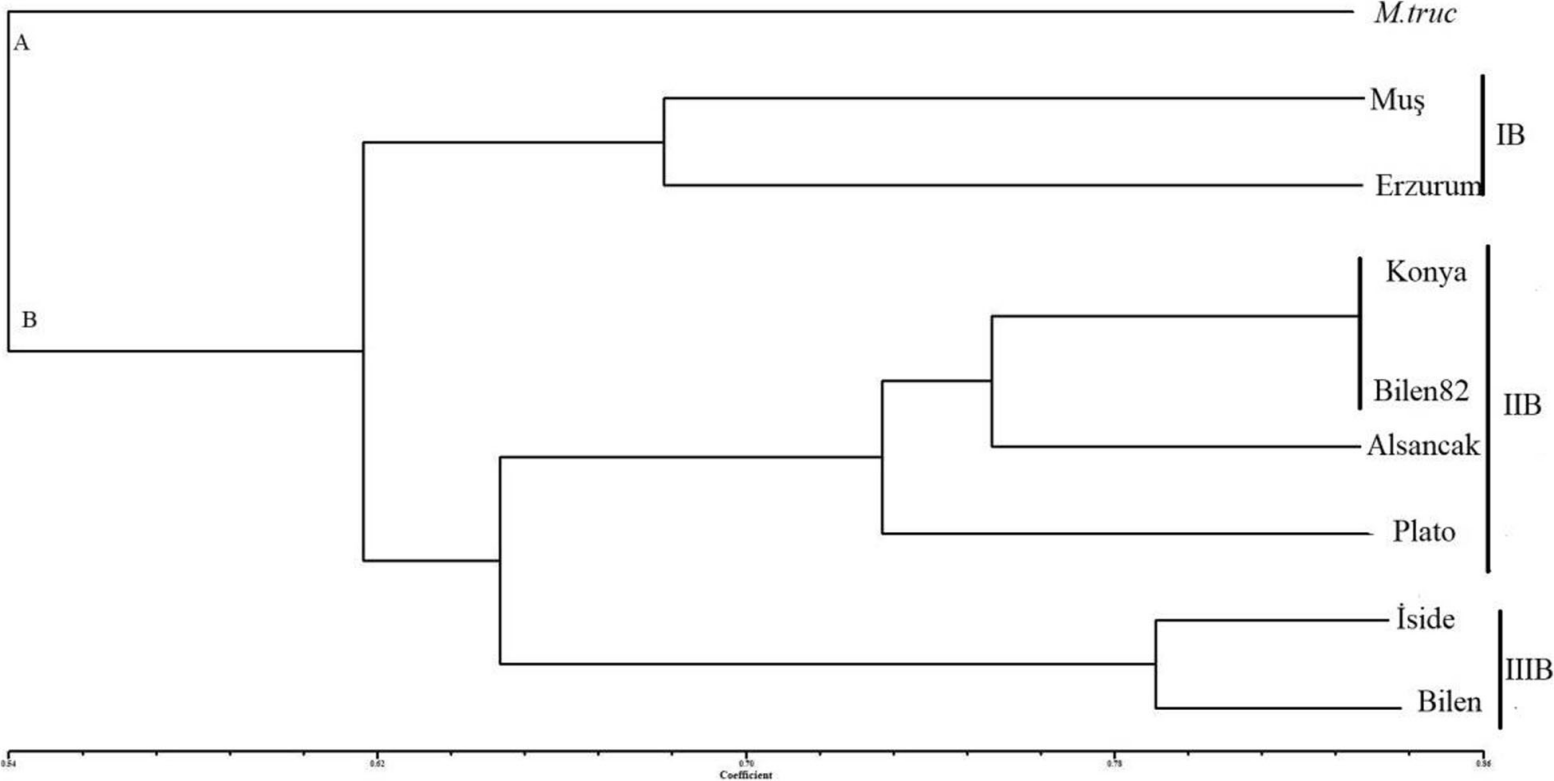
Dendrogram involving genetic diversity between 5 *alfalfa* cultivars, 3 ecotypes, and control (*M*. *truncatula*)

From the *alfalfa* marker databases, we chose 11 SSR markers with transferability present on the genomes of *Medicago truncatula* [17]. The 11 SSR markers successfully generated 50 bands resulting in 22 alleles on the genomes of 8 *alfalfa* varieties and 1 control plant. The PIC values ranged from 0.1800 (*mtic238*) to 0.3700 (*mtic248*). Therefore, the greatest genetic knowledge was obtained from *mtic441* and *mtic471*, while the least was from *mtic238-mtic248* (Table 3). The highest numbers of polymorphic bands (17) were generated from *mtic230*, while the lowest (1 for each) were obtained from *mtic238* and *mtic248*. Using 11 SSR markers, the observed number of alleles, effective number of alleles, gene variation values, Shannon’s information index, and number of total bands were also assessed for the varieties plants used in this study. According to those values, the least value was observed from primers *mtic238* and *mtic248* while the greatest was observed from *mtic441 and mtic471* while the lowest was from *mtic238* (Table 4). In terms of similarity matrix, we constructed a dendrogram in which similarity coefficients ranged from 0.32 to 0.85 for all varieties (Table 3). The dendrogram was composed of two main groups in which *M*. *truncatula* was separated from the *alfalfa* varieties (Fig. 3). In the first main group (B), varieties were in the IB subgroup composed of *Muş* and *Erzurum* ecotypes. In the second, varieties were in the IIB composed of varieties *Konya*, *Bilensoy82*, *Alsancak*, and *Plato*. In the last subgroup, varieties were in the IIIB composed of *Iside* and *Bilensoy* (Fig. 3).

**Table 3:** Genetic similarity for 9 varieties of *alfalfa*

	Mt	*Muş*	*Konya*	*Erzurum*	*Plato*	*İside*	*Bilensoy*	*Bilen82*	*Alsancak*
Mt	1								
*Muş*	0.3214	1							
*Konya*	0.6071	0.7143	1						
*Erzurum*	0.4286	0.6786	0.6071	1					
*Plato*	0.5714	0.6071	0.6786	0.5000	1				
*İside*	0.5000	0.4643	0.5357	0.6429	0.5000	1			
*Bilensoy*	0.6429	0.6786	0.6786	0.7143	0.7143	0.7857	1		
*Bilen82*	0.6786	0.5714	0.8571	0.6786	0.7500	0.6786	0.7500	1	
*Alsancak*	0.5357	0.5714	0.7143	0.6071	0.7500	0.6071	0.6786	0.7857	1

**Table 4:** Different genetic variations estimates for 11 transferability primers used in this study

Locus	Sample size	na*	ne*	h*	I*	PIC
*mtic48*	9	2	4.4707	0.8518	1.3864	0.2500
*mtic64*	9	2	2.01035	0.37035	0.6136	0.2350
*mtic77*	9	2	3.6372	0.7571	0.4983	0.2433
*mtic93*	9	2	6.3019	1.3036	2.0202	0.2620
*mtic230*	9	2	9.5003	1.9963	3.0482	0.2771
*mtic232*	9	2	1.8	0.4444	0.6365	0.3500
*mtic238*	9	2	**1.2462**	**0.1975**	**0.3488**	**0**.**1800**
*mtic248*	9	2	**1.2462**	0.3362	**0.3488**	**0**.**1800**
*mtic441*	9	2	**1.9756**	**0.4938**	**0.6870**	**0**.**3700**
*mtic451*	9	2	2.234	0.4444	0.6923	0.2750
*mtic471*	9	2	**1.9756**	0.4938	0.6870	**0**.**3700**
Mean	9	2	**1.5445**	**0.3263**	**0.4983**	0.2720
St. dev	9	0	0.3206	0.1319	0.1513	

Observed number of alleles (na*), effective number of alleles (ne*), Nei’s (1973) gene diversity (h*), and Shannon’s information index (I*) values

### Total proteins analysis

**Fig. 4:**
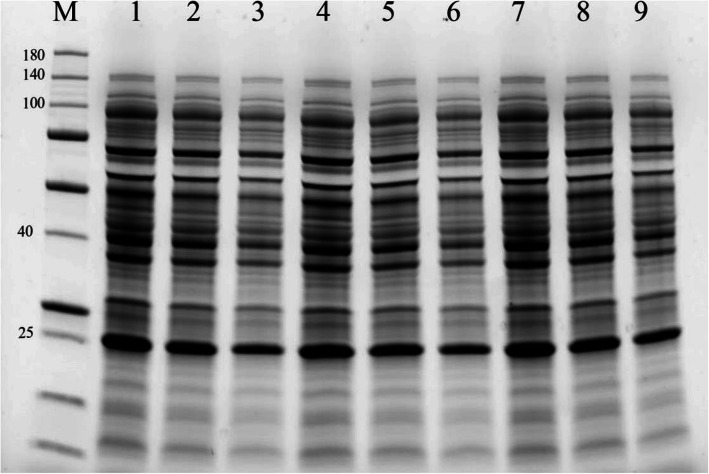
SDS PAGE profiles of total proteins from 5 *alfalfa* varieties, 3 ecotypes, and control (*M*. *truncatula*). M, marker (kDa); 1: *M*. *truncatula*, 2: *Bilensoy*, 3: *Plato*, 4: *Alsancak*, 5: *İside*, 6: *Bilensoy82*, 7: *Konya*, 8: *Erzurum*, 9: *Muş*

Total protein isolated from leaves of 8 varieties were subjected to 10% SDS PAGE analysis. The amounts of total protein molecular weight were observed in all tested samples at 40 kDa expected sizes. According to the band intensity results, the highest protein levels were observed for *M*. *truncatula*, *Alsancak*, and *Konya* varieties. The lowest protein levels were observed for *Plato* and *Bilensoy82* varieties (Fig. 4).

## Discussion

**Fig. 5:**
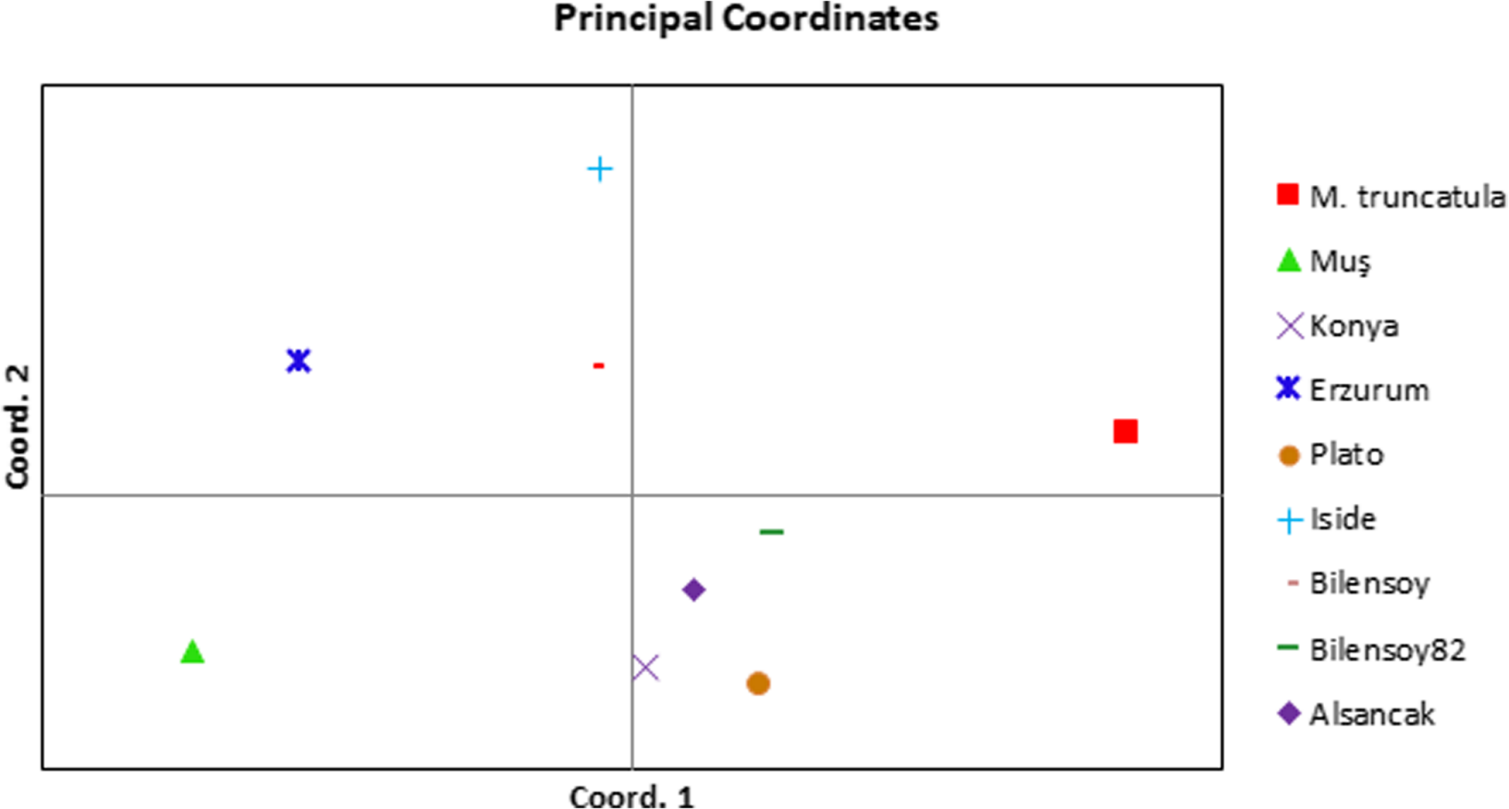
The first two PC plots clarified 53% of total genetic variance with PC1 = 28% and PC2 = 25%

Genetic variations and conservations are vital for economically important crops such as *M*. *sativa* L., *onobrychis*, and *aegilops* [1, 21]. The high variability of *M*. *sativa* L. species and the events of inter- and intra-specific diversity result in the expansion of its genetic variations. However, the identification of species based on agronomic traits can be misleading and might cause complexities during evaluation of species data, taxonomic definition, multiplication, and germplasm conservation. Definition of accurate species is needed not only for taxonomical studies but also to allow the selection of closely related species for the introgression of morphological traits into *Medicago* species [22]. Turkey, along with central Asia, has been discovered as the primitive center of origin for *alfalfa* species due to extensive gene exchange between wild species of *M*. *sativa* L. *varia* and *falcata* species with the same chromosome number *2n = 4x = 32*. Northeastern of Turkey has also been referred as the primary center of diversity for diploid plants. Turkish representatives of the *alfalfa* species contains purple flowers, coiled pods, yellow flowers, uncoiled pods, and intermediates and recombinants between purples flowers and yellow flowers [23]. Genetic variation and gene exchange are important markers for the determination of population genetic structure of natural population region [24]. In many regions of Turkey, highly polymorphous populations of *alfalfa* which came in various forms because of diploid and tetraploid varia cultivated adjacent to each other were encountered. Triploid varieties of *alfalfa* which came into presence through some gene flow between diploids and their tetraploids in Turkey are rarely found. Therefore, we investigated the relation between molecular markers, nuclear DNA contents, and ploidy levels in eight *alfalfa* varieties commonly grown in Turkey using comprehensive techniques. Ploidy levels in plants have been traditionally confirmed by chromosome numbers of stained root tips using microscopy; however, this method is time consuming, occasionally misleading, and insufficient for providing accurate data. Research in genetic variations in the ploidy level of *alfalfa* has been reported within accessions [1, 25]. In our first experiment, the tested varieties exhibited different levels of ploidy within accessions. Chromosome counting analysis demonstrated different chromosome number as diploid, triploid, or tetraploid with some deviations from these levels. A few studies that have shown deviated chromosome numbers were reported for *Medicago* species. These results are similar with one of the reports on Tunisian *alfalfa* population, *Medicago sativa subsp*. *sativa*, and *Medicago sativa varia* [1, 23]. However, the expected basic number was constantly observed in annual *Medicago s*pecies from Algerian Fyad-Lameche et al. [26]. Chromosome number alterations between species of *Medicago* are critical to limiting crossing and gene flow by traditional hybridization. The deviation of expected chromosome numbers can be explained by aneuploidy, which plays an important role in genome evolution. Our results suggest that such relations among varieties indicate that the deviation numbers enclose 30 chromosomes instead of 32 chromosomes. For example, the *Erzurum* and *Konya* varieties were demonstrated to have similar levels in ploidy with basic chromosome number *2n = 4x = 30* (Fig. 1g, h). A count of *2n = 4x = 30* for two varieties probably is the outcome of aneuploid reduction, which presumably is the most common type by deletion of single or multiple chromosome. Aneuploid forms were also observed for *alfalfa* [23]. Similarly, the levels of chromosomes numbers affect not only hybridization but also its physiological and phenotypic traits. They intercross easily and generate viable fertiles because of their similar ploidy levels. These findings clearly indicate the type of aneuploid observed in this study. The two obtained varieties with chromosome number found to be *2n = 4x = 30* is in agreement with the finding of Lapina et al. [27]. Similar results were observed for the outcrossing and gene flow among different *alfalfa* varieties. It is likely that the level of ploidy is associated with the decreasing dysploidy of gene flow. Our observation in *alfalfa* suggests that the former might have derived the latter by reciprocal translocations, which resulted in most of the gene flow of one chromosome to transfer to another. In cytological studies, chromosome information is the main factor and helpful in supporting various species levels. Only *Bilensoy82* was found to be triploid by traditional chromosome analysis among tested varieties (Fig. 1d). This variety is thought to be produced by crossing between *2n = 2x = 16* and *2n = 4x = 32*. Triploid *alfalfa* varieties are less frequent. It increased through gene flow after hybridization of the unreduced gametes from the *2n = 2x = 16* to *2n = 4x = 32* in the genus and have strong male sterility due to chromosomal unbalance. *Bilensoy82* was probably derivated through the formation of one unreduced gamete by a diploid parental plant being from same geographic region in Turkey. The encountered triploid chromosome numbers have been reported, and observations are also consistent with the results found in the genus *Medicago* of northeastern Turkey by Sandoval et al. [7] and Small et al. [23]. Sandoval et al. [7] reported that the hybridization between different chromosome levels are accomplished only about 1% of the time in generating hybrids, the progeny mainly being tetraploids and rare triploids. Gene flow directly indicates the existence of viable reduced gamete formation among diploid and tetraploid *alfalfa*. Our results confirm that this is an extensive gene flow among individual plants at the same or different chromosome levels of *alfalfa* complex in Turkey. Flow cytometry analysis was performed to obtain clearer results of the genome size of samples including *Hordeum vulgare* “Cervios” as the reference plant. Nuclear DNA content and ploidy level in *alfalfa* are complex traits, and flow cytometry is being offered as a powerful new tool for assessing those traits in a fast and inexpensive manner [1, 28]. The estimated results verify that the nuclear DNA content of the tested eight varieties is only slightly different from the biggest genome size of 3.92 pg in the varieties *Plato* obtained in the variety and the smallest nuclear DNA content of 3.71 pg C DNA in the variety *Bilensoy82* which results in an average of 3.81 pg C DNA at 2C DNA per nucleus (Table 2and Fig. 2). A similar value was detected among tested varieties; we did not observe the deviations from the expected nuclear DNA content; thereby verifying the tetraploidy of the tested varieties. Nuclear DNA content estimation revealed that *alfalfa* species have very small genomes and this trait enables the classification of all varieties. However, there was small alteration in the amount of nuclear DNA content in tested varieties that could be related to the growth conditions and geographical region. These results are very similar determination of nuclear DNA content variation among different date palm cultivars (*Phoenix dactylifera L*.) by flow cytometry. The lower nuclear DNA content were obtained from the *Bilensoy82*, *Erzurum*, and *Alsancak* (3.71 pg, 3.75 pg, 3.79 pg ), whereas the higher nuclear DNA content *Plato*, *Bilensoy*, and *Iside* had a nuclear DNA content of 3.92 pg, 3.87 pg, and 3.85 pg, representing a minimal difference in nuclear DNA content among the tested varieties. The reports are different from those of Şakiroğlu et al. [29] who detected that the 2C DNA genome size of *M*. *sativa subsp*. *varia* ranged from 2.85 pg to 4.9 pg. The large variation value could be a result of different sub-species within one species. In contrast, values of nuclear DNA content were tightly clustered to 3.92 pg to 3.71 pg in our study. Additionally, we obtained three peaks for four (*Konya and Muş*, *Iside*, and *Plato*) due to endoreduplication events (Fig. 2). Many studies show that endoreduplication is linked to the growth and content of nuclear DNA for the increase of crop yield [30–32]. The *Fabaceae* is reported as a family of species such as *Medicago*, *Trifolium*, and *Lotus* experiencing endoreduplication events quite frequently. Endoreduplication can be influenced by a number of factors including family affiliation, organ type, life cycle, and ploidy level. However, the genome size of a species is one of the other factors with minor effects. Endoreduplication may accelerate the size increase of heteromorphic species and compensate for small genomes [33]. The content of nuclear DNA was the highest in *Plato* (3.92 pg) and had almost similar values with *Iside* (3.85 pg), *Muş* (3.81 pg), and *Konya* (3.80 pg), whereas all other varieties had low nuclear DNA content. Barow et al. [31] revealed that an inverse correlation exists between the endoreduplication and nuclear DNA content and, in most statement, the varieties with a small genome display higher levels of endoreduplication. Our findings partially confirm this phenomenon; *Muş* variety displayed the highest peak value in endoreduplication followed by *Iside*, *Konya*, and *Plato*. These findings are very similar with one of the first reports on identification and on endoreduplication of *lotus* species [34]. Brummer et al. reported the chromosome numbers and nuclear DNA contents of 263 *alfalfa* and 20 *Medicago falcata* plant species using chromosome counts and flow cytometry. The outcome of the two different methods where ploidy identifications were performed by flow cytometry and traditional chromosome count differed. These results are in agreement with our findings, which demonstrated that flow cytometry is inexpensive and fast on ploidy identification of *alfalfa*. In fact, the results of this study on chromosome analysis and ploidy level are also in agreement with the study on determination of Latvian *alfalfa*. In their study, although the performed flow cytometry count displayed non-variability in nuclear contents between plants of the same accessions, the different chromosome count cells as diploid, triploid, and tetraploid were found in the cultivars of *Skrīveru* and semi wild of *Aizkraukles* and *Dzelmes* [27]. Since root cell existence of endoreduplication is well known, therefore, chromosome numbers in roots and leaves could differ [25]. All tested varieties were found to be tetraploid by flow cytometry analysis. *Alfalfa* is extremely allogamous; thus, it can receive new traits from other species and subspecies. Total protein profile, banding pattern, and cluster research are some of the ways used to evaluate genetic variation in either normal or stress conditions of plants [35]. We suggest that total protein bands be considered to explain the taxonomy specific to the *Medicago* group and other species. Highly variable banding pattern were observed in these tested samples. As observed through total protein analysis, the amount of protein in the tetraploid plant is correlated to the ploidy level. Moreover, *Erzurum* and *Konya* varieties were also almost the same varieties in terms of their protein bands. Interestingly, even *Plato* was considered as tetraploid with respect to cytological test in which the same varieties exhibited the lowest level of protein. Even though data on the protein profiles of perennial *alfalfa* is not available, one contrast result found was the total protein profile for 13 samples on liquid media in annual *Medicago* species of Algerian by Fyad-Lameche et al. [26] because each sample was considered by specific bands. Similar results were obtained using SDS PAGE methods the protein patterns of 47 *Centaurea* species from Turkey. Their results indicate that the SDS PAGE is a useful selection method for determining intra-species degree within *centaurea* [36]. In the present study, all of the amplified primers generated bands and distinguished *Alfalfa* varieties. All transferred markers amplified within *M*. *sativa* L. species were polymorphic. Moreover, transferred primers were performed to observe genetic variation within the genus *alfalfa*. The level of polymorphism was convenient for dendrograms that depicted the genetic similarities of the different *alfalfa* varieties. The dendrogram demonstrated that *alfalfa Muş* variety is closely related to *Erzurum*, but *Konya* variety is distantly related to *Erzurum* and *Muş* varieties (Figs. 3 and 5 and Table 3). *Erzurum* and *Muş* varieties were found to be monophyletic species. One variety (*Konya*) was also found to be monophyletic with *Bilensoy82*, *Muş*, and *Erzurum* varieties were more similar than *Konya* variety, since these two varieties were collected from regions in Turkey closely located to each other. A distant relationship was observed between varieties of *alfalfa* collected at different locations as shown in *Konya* variety (Fig. 5). *M*. *truncatula* was found to be polyphyletic from other varieties and did not show any specific correlation with the populations of *alfalfa* in Turkey, thus verifying the tetraploid nature of the tested varieties, which is also confirmed by flow cytometry peaks (Fig. 3). The observed variations may be expressed by the response of the varieties to the different soil structure, environmental conditions, and gene exchange from other varieties where they were grown. Our results are in line with those of Laamari et al., [37] who verified that the 2C DNA amount of *alfalfa* Gabsi ranged from 2.87 to 3.12 pg. Therefore, they are suggested for identification of accessions of this genus. Julier et al. [8] displayed similar findings when transferring SSR primers used in *M*. *truncatula* to *alfalfa*. The results of SSR also allowed the establishment of similarity among accessions, reflected by genetic similarity estimation and results of cluster analysis.

## Conclusions

This report is one of the recent works involving the cytologic traits, molecular characterization, and protein profile of the *alfalfa* cultivars and ecotypes grown in Turkey. Based on the results, the present work offers rapid and efficient methodology clarifying the genetic variations of *alfalfa* and revealed sufficient genetic variations among the tested eight cultivars and ecotypes. The cytological and molecular techniques used in this study seem to be beneficial in the genetic studies of *alfalfa*. This work provides important findings for the classification, conservation, and innovation of *alfalfa* germplasm resources.

## Data Availability

Not applicable

## Abbreviations

SSR: Simple sequence repeat
AFLP: Amplified fragment length polymorphism
RAPD: Randomly amplified polymorphism DNA
SDS: Sodium dodecyl sulfate
